# Modulation of neural circuits underlying temporal production by facial expressions of pain

**DOI:** 10.1371/journal.pone.0193100

**Published:** 2018-02-15

**Authors:** Daniela Ballotta, Fausta Lui, Carlo Adolfo Porro, Paolo Frigio Nichelli, Francesca Benuzzi

**Affiliations:** 1 Department of Biomedical, Metabolic and Neural Sciences, University of Modena and Reggio Emilia, Modena, Italy; 2 Center for Neuroscience and Neurotechnology, University of Modena and Reggio Emilia, Modena, Italy; Universita degli Studi di Udine, ITALY

## Abstract

According to the Scalar Expectancy Theory, humans are equipped with a biological internal clock, possibly modulated by attention and arousal. Both emotions and pain are arousing and can absorb attentional resources, thus causing distortions of temporal perception. The aims of the present single-event fMRI study were to investigate: a) whether observation of facial expressions of pain interferes with time production; and b) the neural network subserving this kind of temporal distortions. Thirty healthy volunteers took part in the study. Subjects were asked to perform a temporal production task and a concurrent gender discrimination task, while viewing faces of unknown people with either pain-related or neutral expressions. Behavioural data showed temporal underestimation (i.e., longer produced intervals) during implicit pain expression processing; this was accompanied by increased activity of right middle temporal gyrus, a region known to be active during the perception of emotional and painful faces. Psycho-Physiological Interaction analyses showed that: 1) the activity of middle temporal gyrus was positively related to that of areas previously reported to play a role in timing: left primary motor cortex, middle cingulate cortex, supplementary motor area, right anterior insula, inferior frontal gyrus, bilateral cerebellum and basal ganglia; 2) the functional connectivity of supplementary motor area with several frontal regions, anterior cingulate cortex and right angular gyrus was correlated to the produced interval during painful expression processing. Our data support the hypothesis that observing emotional expressions distorts subjective time perception through the interaction of the neural network subserving processing of facial expressions with the brain network involved in timing. Within this frame, middle temporal gyrus appears to be the key region of the interplay between the two neural systems.

## Introduction

Time processing is crucial for every-day life and is flexibly modulated by ongoing experiences. The temporal perception literature shows that emotions are one of the most significant sources of temporal distortion. Different types of emotional stimuli, such as affective images [1–4], fear-inducing stimuli [5] and emotional sounds [6], have been used to investigate the mechanisms underlying emotional modulation of subjective time experience. In particular, consistent findings have been obtained using emotional facial expressions [7,8]: fearful, angry, happy and sad expressions were perceived as lasting longer than neutral expressions presented for the same duration.

Studies dealing with pain-driven temporal distortions have shown conflicting results. Indeed, both temporal over and underestimation have been reported during painful stimulation. Longer perceived durations for painful than neutral stimuli have been described in animals [9] and in healthy humans using prospective time judgments (time reproduction and production, verbal estimation) during electric shock [10,11] and hot thermal stimulation [12], and in adult [13] and pediatric [14] population of migraineurs. On the contrary shorter estimates have been observed in humans using retrospective time judgments (verbal estimation) during cold thermal stimulation [15] and in patients with chronic headache [16].

In retrospective paradigms, participants are not aware that they will have to estimate the duration of a target until the target is over. Therefore, focusing attention to time is not required to retrospectively assess duration, because retrospective estimates rely on incidental memory for temporal information [17]. On the contrary, prospective time estimates focus on experienced duration [18] and attention to time is essential [17]: participants are informed that they will have to estimate the duration of a target. Prospective paradigms have been largely adopted in order to study emotional temporal distortions which have been interpreted within the framework of the internal clock theory. According to the Scalar Expectancy Theory (SET) [19], timing is achieved via a biological internal clock consisting of a pacemaker that emits pulses, a switch, and an accumulator. The number of pulses collected by the accumulator represents the duration of the interval. The collected pulses are transferred to the accumulator via the switch, which closes at the beginning of the interval to be timed, thus allowing the passage of the pulses, and opens at the end (clock stage). Subsequently, the duration of the interval (the number of collected pulses) can be transferred from working memory to long-term memory (memory stage) to be compared with abstract temporal references (decision-making stage). The rate at which pulses are emitted is sensitive to arousal, with increase in the level of arousal leading to increase in the number of produced pulses and thus in the perceived duration [20–24]. Emotions are arousing and cause overestimation of the interval to be timed; they are therefore thought to affect perceived time by speeding up the pacemaker [25–27]. More arousing facial expressions of emotion, such as fearful and angry expressions, cause larger overestimation of time, compared to less arousing emotions (sadness and happiness) [28]. It has been found also that valence and arousal interact in affecting duration judgments [3]: the duration of unpleasant stimuli was overestimated in high arousal conditions, whereas it was underestimated in low arousal conditions; the opposite effect was found for pleasant pictures. These authors suggest that an attention-driven mechanism may be triggered during low arousal emotional situations. The SET suggests that the amount of attentional resources allocated to time will affect the subjective perceived duration [29], influencing the functioning of the switch. When attention is diverted to non-temporal information at the beginning of the interval, the switch either closes with a longer latency, or it begins to flicker. The resulting underestimation of the duration is due to the loss of some pulses [30].

The interpretation of emotional facial expressions plays an important role in everyday social functioning [31]. In some instances, facial expression of pain has been reported to be specific and distinct from the expressions of basic emotions, thus clearly recognizable as pain-related by observers [32,33]. Pain signals have, indeed, important survival value: demand rapid detection allowing adaptive behavioral responses of the observer [34]. Recent findings have shown that observing painful facial expressions increases cerebral activation within portions of the neural circuit active during the direct experience of pain and within the cerebral network of emotional face processing (fusiform gyrus, superior/middle temporal gyrus, STG/MTG, inferior frontal gyrus, IFG, medial prefrontal cortex, amygdala) [35], both when processing is explicit [36–38] and when it is implicit [39]. To our knowledge, only two neuroimaging studies have examined subjective temporal perception during the observation of socially relevant stimuli, such as aversive/non-aversive pictures [4] and angry/happy faces [40]. Therefore, the neural substrates of the emotional modulation of time perception are poorly understood and further data is needed; studies on facial expressions of pain may be used to improve our knowledge of this issue.

The aims of the present single-event fMRI study were to investigate: a) whether observation of facial expressions of pain interferes with time production; and b) the neural substrates of potential subjective temporal distortions due to concurrent processing of painful expression.

We have explored these issues in healthy volunteers observing facial expressions, either neutral or painful. A dual-task procedure was used: a gender discrimination task and a concurrent temporal production task. We preferred to adopt an implicit pain processing task in order to avoid the differential employment of cognitive resources required for the explicit recognition of emotional expression.

## Materials and methods

### Participants

Thirty right handed volunteers (15 women; mean age 21.7 ± 2.2; range: 19–30; mean education: 13.6 years) with no history of psychiatric or neurological disease took part in the fMRI study. Sample size was chosen according to published guidelines for fMRI experiments in healthy volunteers [41]. The experimental protocol had been approved by the local Ethics Committee of Modena (protocol number 3903/C.E.) and all subjects gave their written informed consent to take part in the study.

### Stimuli validation

In order to offer a real representation of pain expressions, we chose to select the most expressive frames belonging to video clips previously recorded during cutaneous mechanical stimulations in thirty-five volunteers (6 males; mean age 22 ± 2). These subjects were different from the ones participating in the validation phase and in the fMRI experiment (see below). Forty video-clips (20 painful, 20 neutral), each lasting two seconds, were recorded from each volunteer while he/she was receiving painful or tactile (non painful) stimulation to their right hand dorsum or palm by means of a custom-built stimulator. VirtualDubMod (http://virtualdubmod.sourceforge.net/) was used to extract one hundred three frames from the most expressive recorded videos, according to the evaluation of the experimenters. The extracted faces were presented to another group of healthy volunteers (37 healthy participants, 15 male; mean age 28 ± 3; range 24–37), different from the ones who took part in the fMRI session. Twenty-two frames depicted neutral faces, whereas eighty-one frames showed expressions of pain. Each frame was presented twice. At the first presentation of each frame, the volunteers were asked to freely classify the facial expression and to rate the intensity of the expression on a 0–10 point scale (free validation). After the free validation phase was concluded for all the frames, subjects were asked to indicate whether each face was painful or not and to rate how painful each stimulus was on a 0–10 point scale (guided validation). Of all the responses provided during the free validation of pain-related stimuli, 59% was “pain”, 10.5% was “sadness”, 6.4% was “disgust”, 4.7% was “fear”, 0.6% was “anger”, 18.7% referred to heterogeneous descriptions (e.g., “bored”, “fatigued” etc.). The stimuli identified as painful by at least 25% of the subjects in the free validation phase, and by 75% of the group in the guided validation phase, were selected for the fMRI study. Pictures evoking pain evaluation > 1.5 were not included among the neutral stimuli.

### Experimental design

**Fig 1:**
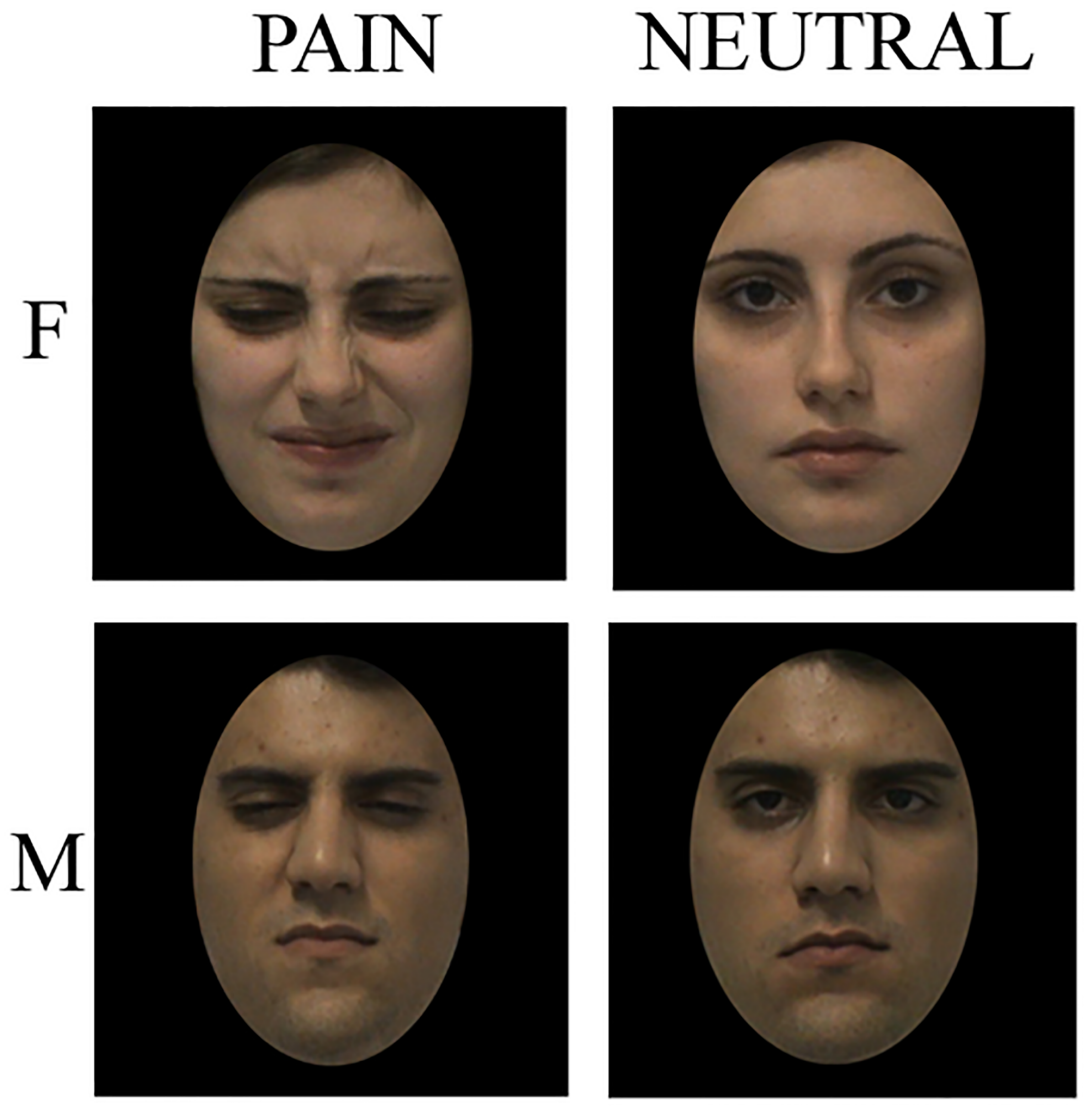
Experimental stimuli. Examples of painful and neutral facial expressions presented during the fMRI temporal production experiment. F = Female, M = Male.

The final series of stimuli administered during the fMRI session consisted of 38 expressions, 27 related to pain (P; mean pain rating obtained in the guided validation phase = 6.02) and 11 neutral (N; mean pain rating obtained in the guided validation phase = 0.6; see Fig 1). Eleven different identities were used (2 males). The presented facial expressions (pain and neutral) were counterbalanced within each volunteer: 50% of the trials were pain, 50% were neutral. The proportion of the presented faces gender (22% male, 78% female) was the same for all volunteers. The individuals whose pictures were included in this manuscript have given written informed consent (as outlined in PLOS consent form) to publish these case details.

The experimental protocol included a training phase, which took place outside the scanner, and a test phase, performed during the fMRI acquisition.

**Fig 2:**
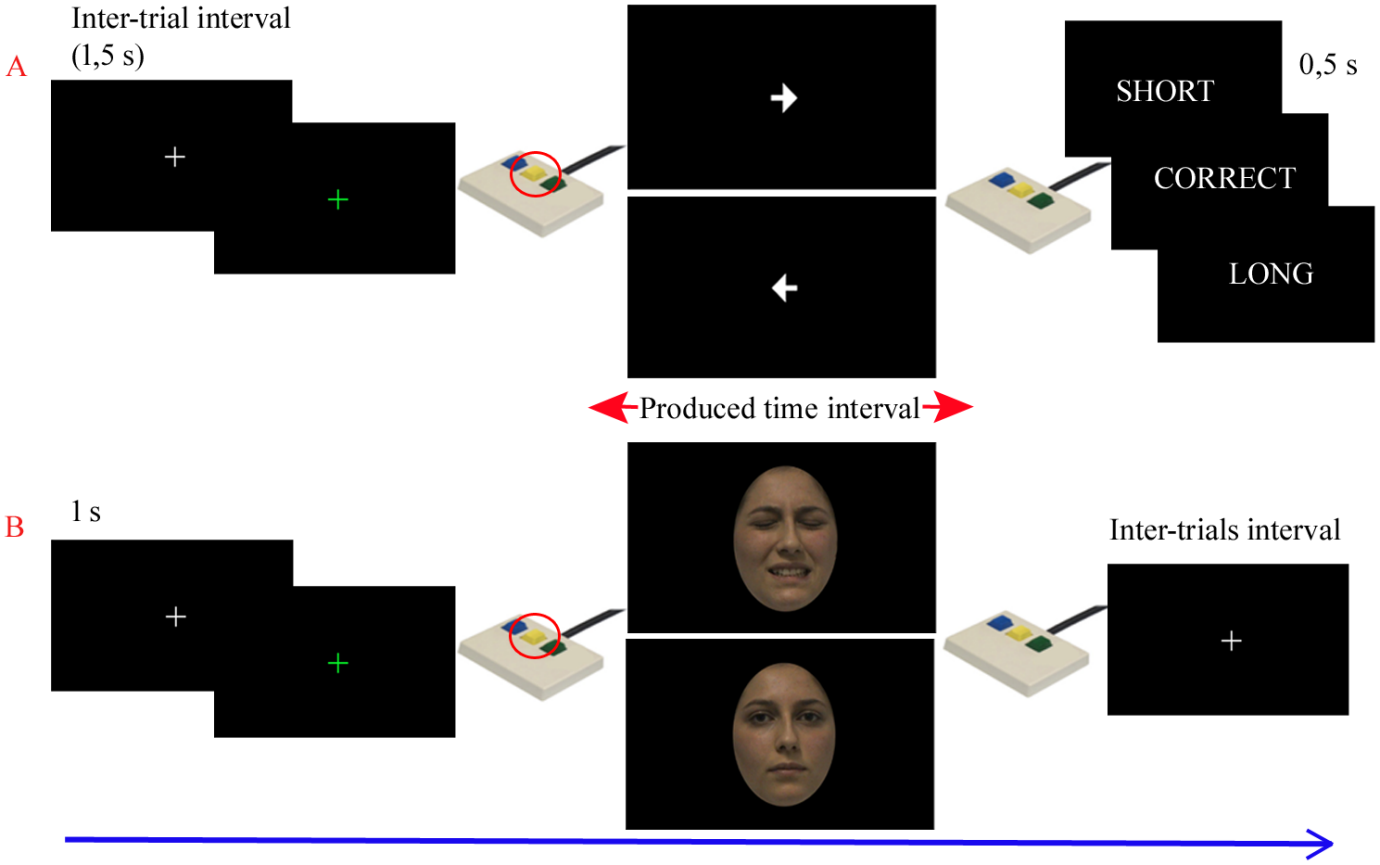
Experimental paradigm. Schema of the training (A) and fMRI (B) sessions. A) During the training session, the following visual stimuli were sequentially presented at the center of the screen: a white cross (1.5 s), a green cross persisting until the central button was pressed, an arrow facing left or right (signaling whether the left or right button had to be pressed to stop the time interval), and a visual/acoustic feedback (500 ms) about the accuracy of the produced interval. B) During the fMRI session, the same sequence was adopted, but with male or female (painful or neutral) faces instead of arrows. During both sessions, production of a 3 s time interval was requested during each trial. No feedback was given on the accuracy of the time intervals during the fMRI experiment.

*Training phase*. During the training phase (Fig 2A), the participants were presented with a green cross appearing at the center of a computer screen. Subjects were asked to produce a 3 s interval by pressing two buttons of a response pad in sequence. The same time interval has been used in previous studies during temporal production/reproduction tasks [3,42] and for a dual-task paradigm [43]. First, the subjects had to press the central button with the right hand middle finger to start the interval; then, depending on the arrow appearing at the center of the screen, the volunteers were requested to press either the left or the right button of the response pad to stop the subjective 3 s interval. This was done to accustom subjects to use either button, as they would be requested to do in the test phase (see below). A visual and acoustic feedback on the accuracy of the temporal production task was presented for 500 ms after each trial. The intervals were considered to be correct when longer than 2,850 ms and shorter than 3,150 ms. The inter-trial interval (ITI) lasted 1,500 ms, during which a white cross appeared on the computer screen. One hundred trials were administered to each participant. For each subject the above mentioned criterion was applied to the median of the produced intervals for the training session in order to verify the improvement in temporal production and to decide whether further training was necessary. Fifty additional trials were administered if the median of the produced intervals didn’t fall within the correct range. The training phase was completed just before (10–15 min) the test phase.

*Test phase*. During the test phase (Fig 2B), subjects were assigned a dual-task, which consisted of a temporal production task and a gender discrimination task, performed concurrently. Therefore, the influence of painful facial expressions on time processing was explored using an implicit task. An event-related fMRI paradigm was used. Each subject performed 3 runs, 24 trials each, for a total of 72 trials. At first, a white cross was presented at the centre of the screen (1 s) followed by a green cross. From the appearing of the green cross, 16 s were available for the participants to produce the subjective interval (3 s). Therefore, each trial lasted 17 seconds. The beginning of the interval and the appearing of the face were defined by pressing the central button of a response pad using the right hand middle finger. Subjects were instructed to watch the face and to press the left button of the response pad (using their right index finger) or the right button (using their right ring finger) to indicate the gender of the face at the end of the subjective time interval. The association between stimulus (male or female) and response (left or right button) was counterbalanced between the subjects. The pseudo-random order of stimuli (faces) was different for each participant. Each stimulus was presented no more than 3 times during the experiment and covered a visual angle of 18°. Two rest blocks lasting 15 and 16 seconds, respectively, were included at the beginning and at the end of each run. At the beginning of each run, in order to isolate the brain activations associated with motor response, the volunteers were asked to press left, right or central button according to the stimulus appearing on the screen (the arrow facing left, right or the green cross, respectively). The green cross was presented six times, whereas the two arrows were presented three times each (in pseudo-random order). The participants were told that these responses were used to check the functioning of the response pad.

During the scanning phase, custom-made software developed in Visual Basic 6 (http://digilander.libero.it/marco_serafini/stimoli_video/) was used to present stimuli via the ESys functional MRI System (http://www.invivocorp.com) remote display, and to collect behavioral responses.

At the end of the scanning session, participants were presented with the same stimuli again and were asked to rate the intensity of the facial expression and how painful each stimulus was on a 0–10 point scale. Finally, two different personality scales were administered to the volunteers: the Interpersonal Reactivity Index (IRI) [44], a self-report rating index developed to measure personal empathy defined as the “reactions of one individual to the observed experiences of another”, and the Pain Catastrophizing Scale [45], a 13-item instrument developed to assess the subject’s level of catastrophic thinking about pain.

### fMRI data acquisition

Functional imaging was performed using a Philips Achieva system at 3T and a gradient-echo echo-planar sequence from 30 axial contiguous slices (repetition time, TR = 2,000 ms; in-plane matrix = 80x80; voxel size: 3x3x4 mm). A total of 756 volumes was acquired over three 8 min 24 s runs per volunteer. A high-resolution T1-weighted anatomical image was acquired for each participant to allow anatomical localization. The volume consisted of 170 sagittal slices (TR = 9,9 ms; TE = 4,6 ms; in plane matrix = 256x256; voxel size = 1x1x1 mm). fMRI data processing was performed using Matlab 7.11 and SPM12 (Wellcome Department of Imaging Neuroscience, London, UK). Functional volumes of each participant were corrected for slice-time acquisition differences, realigned to the first volume acquired, normalized to the MNI (Montreal Neurologic Institute) template implemented in SPM12, and smoothed with a 9x9x12 mm FWHM Gaussian kernel. Sixteen trials were excluded because subjects didn’t respond, whereas the third run of one subject was discarded because of excessive movement.

### Behavioral data analyses

At the end of the training phase, the median of the produced intervals was determined for each volunteer; for all of them, it fell within the accepted interval (2,850–3,150 ms).

Two separate measures were obtained from the dual-task performed during the fMRI acquisition session: the medians of the produced intervals and the coefficients of variation (standard deviation /mean). Two separate ANOVAs were made to compare these measures using the factors Condition (P, N) and Run (1, 2, 3). In order to minimize type I statistical errors, we decided to use a conservative *post hoc* test (Scheffé’s test) for statistical comparisons.

A *t*-test was used to compare the post-scanning evaluation of perceived pain of P and N stimuli.

Free online software (https://www.psychometrica.de/effect_size.html) was used to estimate the effect size (Cohen’s d) of the ANOVAs and *t*-tests.

Finally, linear regressions were used to explore the relation between the medians of the produced time intervals during the fMRI experiment and the individual personality trait assessed in the post-scanning questionnaires (IRI and PCS).

### GLM analysis

At first, functional data from each volunteer were analyzed individually by means of the SPM12 general linear model (GLM). Two different analyses were performed in order to emphasize temporal processing (analysis 1) or observation of painful expressions (analysis 2).

Analysis 1: fMRI activity changes related to temporal production. Two types of conditions were used: pain (P) and neutral (N). Trials were classified according to the a priori categorization of the stimuli as painful or neutral. Stimulus duration corresponded to the subjectively determined interval in each trial. The main weighted contrasts of interest were “P+N > motor response” and “P > N”;
Analysis 2: fMRI activity changes related to the observation of painful faces during temporal production. Subjectively determined onset and end in each painful and neutral trial were considered separately in this analysis. Events were therefore classified as P-onset (Po), P-end (Pe), N-onset (No), N-end (Ne). Considering that faces are perceived in the first 120 ms after they are presented [46], events were modeled as instantaneous. The contrast of interest was “Po > No”.

The design matrices contained the experimental conditions and the motor response (referring to the initial response pad test) as regressors of interest, and the 6 motion correction parameters as confounds. Condition-specific effects were estimated according to the GLM and compared using linear contrasts.

The resulting contrast images were entered in the second-level random effect analyses.

### PPI analysis

In order to investigate task-specific functional interactions between cortical areas, two different psycho-physiological interaction (PPI) analyses [47,48] were carried out. The first PPI used the supplementary motor cortex (SMA) cluster resulting from “P+N vs. motor response” GLM contrast in Analysis 1 (see Results) as seed. A second PPI analysis was implemented using the right MTG identified in the “Po > No” conventional GLM contrast in Analysis 2 (see Results) as seed. Recent findings suggest that the SMA may be part of the putative clock mechanism as temporal accumulator [49], whether the MTG has been previously reported as active during emotional face perception [35,50,51] and the presentation of painful facial expressions [38].

In each participant, the signal from the peak voxel in SMA was extracted from the contrast “P > N”, whereas the map of the contrast “Po > No” was used to extract the peak voxel activation in MTG. A 6-mm-radius sphere was built around the activity peak to define a volume of interest (VOI; MNI average coordinates: SMA: x = -2.2, y = -0.4, z = 62.8; MTG: x = 59.7, y = -54.7, z = 2.4). Each participant’s data were re-modeled with regressors for: the time-course in the seed region (*physiological regressor*); the experimental condition (vision of painful versus neutral faces during temporal production; *psychological regressor*); the interaction between the experimental condition and the region of interest activation signal (*psychophysiological interaction*, PPI). The latter was chosen as the regressor of interest and the corresponding contrast images of the single-subject PPI analyses were used for the random-effect analyses. A one sample *t*-test was utilized.

Finally, regression analyses were implemented to explore which brain regions showed a correlation with the median of the produced intervals.

For all analyses, except for “P+N vs. motor response” contrast, a double statistical threshold (voxel-wise p < 0.001 and spatial extent) was adopted to achieve a combined significance, corrected for multiple comparisons, of α < 0.05, as assessed by 3dClustSim (https://afni.nimh.nih.gov/pub/dist/doc/program_help/3dClustSim.html). For the contrast “P+N vs. motor response”, a Family Wise Error (FWE) correction was used. The Matthew Brett correction (mni2tal: http://www.mrc-cbu.cam.ac.uk/Imaging/mnispace.html) was applied to the SPM-MNI coordinates to obtain the coordinates in Talairach space [52].

## Results

### Behavioural data

For all the following data, median ± standard deviation (SD) are provided.

During the training session, the volunteers produced a median time interval of 2,985 ± 61.8 ms. ANOVA on the medians of the produced intervals during the fMRI experiment (dual-task) showed a significant effect of the factor Condition (F_(2,87)_ = 5.13; p < 0.05); *post hoc* test showed that the volunteers produced longer intervals in the P condition (3,372 ± 596 vs. 3,333 ± 550; p < 0.05; d = 0.38). No significant effect of the factor Run (F_(1,87)_ = 0.02; p = 0.98) nor of the Condition x Run interaction (F_(2,87)_ = 2.2; p = 0.12) were found. ANOVA on the coefficients of variations didn’t show any significant effect of the factors.

In the post-scanning evaluation, the mean intensity (6.4 ± 1.3 vs. 1.4 ± 0.9; t_(29)_ = 22.3; p < 0.001; d = 5.18) and perceived pain (6.06 ± 1.3 vs. 0.5 ± 0.6; t_(29)_ = 20.4; p < 0.001; d = 6.3) ratings were significantly different between painful and neutral stimuli, corroborating the results of the stimuli validation.

No significant correlation was found between the medians of the produced time interval and the individual scores in either personality trait, considering all the subscales of IRI (r < -0.26; p > 0.16) and PCS (r < -0.35; p > 0.06) data.

### GLM analysis

#### Analysis 1: fMRI activity changes related to temporal production

**Fig 3:**
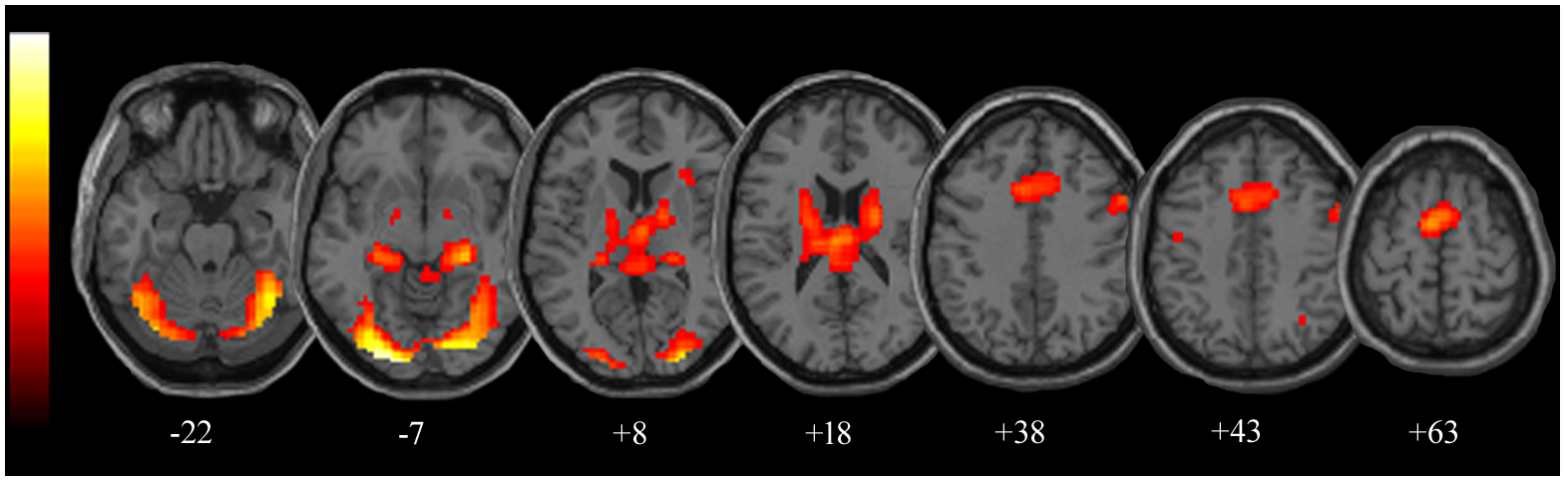
fMRI activity changes related to temporal production. Results of the “P+N vs. motor response” contrast, shown on axial slices of a standard structural T1 weighted brain image (p < 0.05 FWE corrected, k ≥ 10).

The contrast “P + N vs. motor response” (Table 1; Fig 3) revealed activity in areas previously reported to play a role in temporal processing [49,53–57], including SMA bilaterally, right anterior insula (AI), right IFG and angular gyrus, middle cingulate cortex (MCC), and bilaterally in cerebellum, hippocampus and parahippocampal gyrus. Activated foci were also found bilaterally in fusiform gyrus and in middle occipital gyrus. Subcortical activations were found bilaterally in thalamus, putamen and caudate nucleus (body and tail).

**Table 1:** fMRI activity changes related to temporal production (results of the “P+N vs. motor response” contrast; p < 0.05 FWE corrected, k ≥ 10 voxels).

				*Spatial Coordinates*					
				*MNI*			*Tal*		
*Anatomical regions*	*Side*	*k*	*Z*	*x*	*y*	*z*	*X*	*y*	*Z*
Middle occipital gyrus (BA 17, 18), fusiform gyrus (BA 19, 37), cerebellum	L/R	1469	> 8	-27	-88	-10	-27	-86	-4
			> 8	-15	-94	-10	-15	-91	-4
			> 8	27	-91	2	27	-88	6
Hippocampus, parahippocampus, thalamus, caudate nucleus (body and tail), putamen	L/R	1002	7.22	27	-28	-2	27	-27	0
			6.76	-27	-31	-2	-27	-30	0
			6.21	-3	-19	18	-3	-18	17
Supplementary motor cortex (BA 6), mid-cingulate gyrus (BA 24, 32)	L	382	6.50	-3	-1	66	-3	2	61
			5.79	-3	11	46	-3	13	42
			5.68	-6	17	38	-6	18	34
Amygdala, hippocampus, parahippocampal gyrus (BA 34, 28)	L	67	5.71	-18	-4	-14	-18	-4	-12
Middle and inferior frontal gyri (BA 9)	R	50	5.68	57	11	34	56	12	31
			5.35	54	8	42	53	10	38
Anterior insula (BA 13)	R	31	5.32	30	20	14	30	20	12
Middle frontal gyrus (BA 6)	L	32	5.27	-48	-7	50	-48	-4	46
			4.84	-45	2	54	-45	4	50
Angular gyrus (BA 39)	R	11	4.95	30	-61	46	30	-57	45
Amygdala, parahippocampal gyrus (BA 34)	R	19	4.82	18	-1	-18	18	-2	-15

BA = Brodmann Area, R = right, L = left

#### Analysis 2: fMRI activity changes related to the observation of painful faces during temporal production

**Fig 4:**
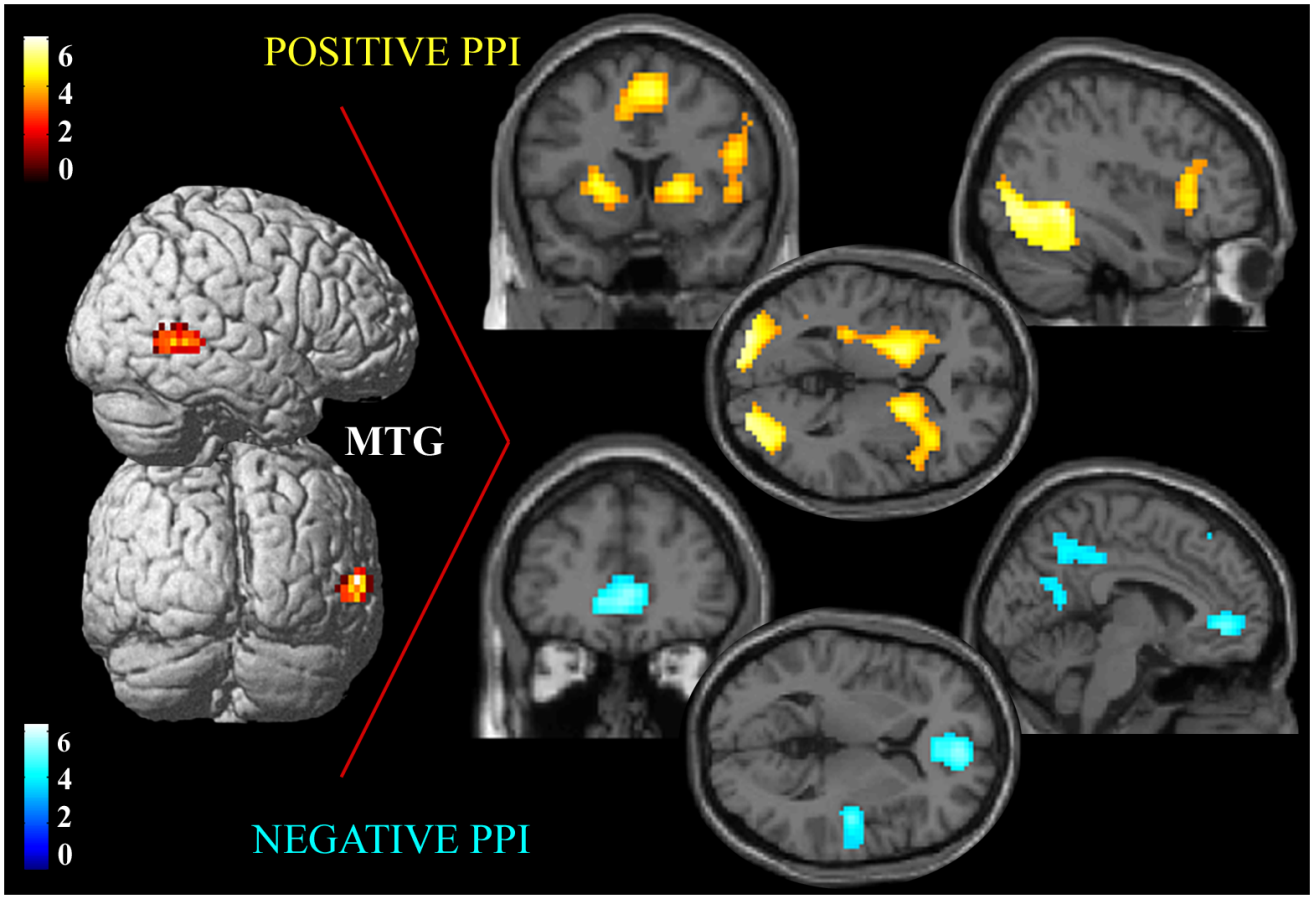
fMRI activity changes related to the observation of painful faces during temporal production and MTG connectivity. Results of the PPI between neural activity in the right middle temporal gyrus (MTG) and the psychological variable of interest (implicit observation of pain expression during temporal processing). Left: Focus of activity in the right MTG related to the presentation of P faces, superimposed on a surface rendering of the brain. Right: positive (top) and negative (bottom) PPI between neural activity within the right MTG and the psychological variable of interest; foci are shown on sagittal, coronal, and axial slices of a standard structural T1 weighted brain image. A double statistical threshold was adopted to correct for multiple comparisons, α < 0.05 (3dClustSim): voxel-wise intensity of p < 0.001, and k > 64 voxels, for GLM analysis, k > 70 voxels, for PPI analyses.

The contrast “Po vs. No” showed the activation of the posterior portion of right MTG (Table 2; Fig 4).

**Table 2:** fMRI activity changes related to the observation of painful faces during temporal production (results of the “Po vs. No” contrast; 3dClustSim correction for multiple comparisons, α < 0.05: voxel-wise intensity threshold of p < 0.001, k > 64 voxels).

				*Spatial Coordinates*					
				*MNI*			*Tal*		
*Anatomical regions*	*Side*	*k*	*Z*	*x*	*y*	*z*	*X*	*y*	*Z*
Middle and superior temporal gyri (BA 21, 22)	R	70	3.99	60	-55	2	59	-53	5
			3.97	60	-43	2	59	-42	4

BA = Brodmann Area, R = right, L = left

### PPI analysis: SMA connectivity

**Fig 5:**
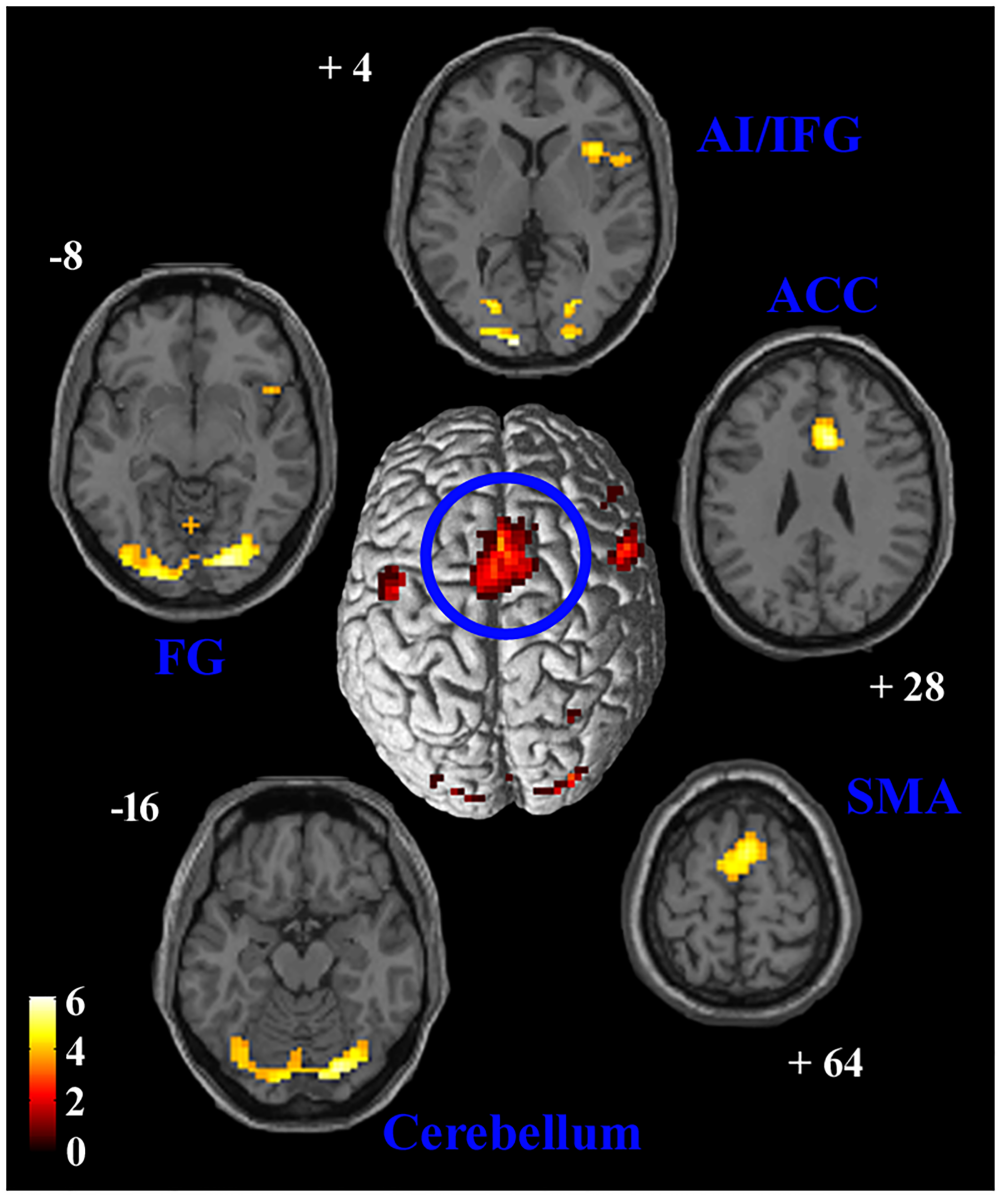
SMA connectivity. Results of PPI between neural activity in the SMA and the psychological variable of interest (implicit observation of pain expression during temporal processing). The SMA cluster of activation used as seed is displayed at the center, superimposed on a surface rendering of the brain. Areas of significant positive PPI are shown in axial slices of a standard structural T1 weighted brain image. A double statistical threshold was adopted to correct for multiple comparisons, α < 0.05 (3dClustSim): voxel-wise intensity of p < 0.001, and k > 69 voxels. FG = fusiform gyrus, SMA = supplementary motor area, ACC = anterior cingulate cortex, AI = anterior insula, IFG = inferior frontal gyrus.

Viewing painful expressions during temporal processing modulated SMA functional connectivity with several cortical regions, including anterior cingulate cortex (ACC) and MCC, right AI and IFG, bilateral cerebellum, middle and inferior occipital gyri and fusiform gyrus (Table 3A; Fig 5). Moreover, functional connectivity between SMA and several frontal regions (bilateral orbito-frontal cortex, medial superior frontal gyrus, left ventro-lateral prefrontal cortex), ACC and right angular gyrus, was correlated to the median produced time interval during painful facial expression processing (“P > N” contrast; Table 3B).

**Table 3:** SMA connectivity changes when comparing painful and neutral faces observation during temporal processing (A; 3dClustSim correction for multiple comparisons, α < 0.05: voxel-wise intensity threshold of p < 0.001, k > 69 voxels) and correlation between the SMA activity and the median produced time interval (B; 3dClustSim correction, α < 0.05: voxel-wise intensity threshold of p < 0.001, k > 68 voxels).

					*Spatial Coordinates*					
					*MNI*			*Tal*		
	*Anatomical regions*	*Side*	*K*	*Z*	*X*	*y*	*z*	*x*	*y*	*z*
**A**	Middle and inferior occipital gyrus (BA 17, 19), fusiform gyrus (BA 37), cerebellum	L	761	4.65	-9	-97	2	-9	-94	7
				4.55	-12	-82	-22	-12	-80	14
				4.47	-18	-94	-2	-18	-91	3
	Middle and anterior cingulate cortex (BA 24, 32), supplementary motor area (BA 6)	L/R	363	4.63	6	20	38	6	21	34
				4.15	6	8	66	6	11	60
				3.95	-6	2	62	-6	5	57
	Anterior insula (BA 13), inferior frontal gyrus (BA 44)	R	108	3.95	36	17	10	36	17	8
				3.86	54	11	22	53	12	20
				3.42	45	14	-2	45	13	-2
**B**	Medial superior frontal gyrus (BA 10), medial orbito-frontal cortex (BA 11), anterior cingulate cortex (BA 32)	L/R	388	4.26	6	56	26	6	55	21
				4.11	12	56	-6	12	54	-8
				4.04	-15	53	-2	-15	51	-4
	Inferior frontal gyrus, ventrolateral pre-frontal cortex (BA 47)	L	102	3.87	-39	35	-10	-39	33	-10
				3.63	-51	32	-10	-50	31	-10
				3.49	-45	44	-6	-45	42	-7
	Angular gyrus (BA 39)	R	86	3.82	39	-55	30	39	-52	30

BA = Brodmann Area, R = right, L = left

### PPI analysis: MTG connectivity

The PPI analysis showed that the neural activity of MTG was related to the activity of the cerebral network subserving time processing during observation of painful vs. neutral faces. In particular, increased activity was found in left primary sensorimotor cortex, right AI and IFG, right MCC, bilateral SMA, cerebellum, putamen, caudate nucleus and globus pallidus. Positive PPI was also found bilaterally in fusiform and middle occipital gyri (Table 4A; Fig 4).

**Table 4:** Right MTG connectivity changes when comparing painful and neutral faces perception during temporal processing (A: Positive PPI; B: negative PPI; 3dClustSim correction, α < 0.05: voxel-wise intensity threshold of p < 0.001, k > 70 voxels).

					*Spatial Coordinates*					
					*MNI*			*Tal*		
	*Anatomical regions*	*Side*	*K*	*Z*	*X*	*y*	*z*	*x*	*y*	*z*
**A**	Middle occipital gyrus (BA 17), fusiform gyrus (BA 19, 37), cerebellum	L	692	4.86	-36	-58	-18	-36	-57	-12
				4.83	-27	-85	-6	-27	-83	-1
				4.75	-15	-94	-2	-15	-91	-3
	Middle occipital gyrus (BA 18), fusiform gyrus (BA 19, 37), cerebellum	R	806	4.75	30	-52	-22	30	-51	-16
				4.71	42	-46	-6	42	-45	-3
				4.64	30	-85	-6	30	-83	-1
	Primary somatosensory cortex (BA 3), primary motor cortex (BA 4), pre-motor cortex (BA 6)	L	205	4.63	-36	-10	62	-36	-7	57
				4.14	-51	-7	46	-50	-5	43
				3.75	-30	-25	50	-30	-22	47
	Supplementary motor area (BA 6), mid-cingulate cortex (BA 24, 32)	L/R	339	4.52	-3	-1	62	-3	2	57
				3.74	12	20	34	12	21	30
	Putamen, globus pallidus	L	311	4.47	-21	-1	6	-21	-1	6
				4.00	-24	-13	6	-24	-12	6
				3.77	-30	-31	2	-30	-30	3
	Putamen, globus pallidus, caudate nucleus, anterior insula (BA 13), inferior frontal gyrus (BA 47)	R	434	4.34	18	5	2	18	5	2
				3.85	36	23	2	36	22	1
				3.82	48	11	22	48	12	20
**B**	Posterior insula (BA 13), primary and secondary somatosensory cortex (S I, S II), superior temporal gyrus (BA 41)	R	289	4.97	48	-19	14	48	-18	14
				4.85	45	-10	14	45	-9	13
				4.30	60	-19	14	59	-18	14
	Superior parietal lobule (BA 5), primary somatosensory cortex (BA 3), precuneus/posterior cingulate cortex (BA 31)	L/R	430	4.86	21	-46	70	21	-41	67
				4.66	27	-37	70	27	-33	66
				4.11	-15	-40	38	-15	-37	37
	Anterior cingulate cortex (BA 24, 32), medial frontal gyrus (BA 10)	R	252	4.74	3	50	2	3	49	-1
	Middle and superior frontal gyrus (BA 8, 9)	L	210	4.28	-21	29	46	-21	30	41
				4.11	-24	14	58	-24	16	53
				4.09	-30	17	42	-30	18	38
	Middle and superior frontal gyrus (BA 8, 9)	R	120	4.24	21	29	42	21	30	37
				3.90	30	23	46	30	24	41
	Middle occipital gyrus (BA 19), angular gyrus (BA 39)	L	95	4.07	-33	-79	38	-33	-75	39
				4.05	-39	-76	30	-39	-72	31
	Precuneus/posterior cingulate cortex (BA 23, 31)	L	103	3.90	-12	-64	22	-12	-61	23
				3.56	-12	-88	26	-12	-84	28

BA = Brodmann Area, R = right, L = left

Foci showing negative correlations with MTG were found in posterior insula, ACC, primary and secondary somatosensory cortex, cortical regions involved in pain perception [58–60], and in cerebral areas crucial for time processing, such as dorso-lateral prefrontal cortex (dlPFC), bilaterally. Superior parietal lobule of the right hemisphere, left middle occipital gyrus, precuneus/posterior cingulate cortex and, bilaterally, superior frontal gyrus (Table 4B; Fig 4), also showed negative correlation.

## Discussion

The main results of this study were as follows: a) as expected, the concurrent gender discrimination task interferes with temporal production [29], causing underestimation of time intervals (namely, participants produced longer intervals); the underestimation was significantly higher when observing facial expressions of pain, as compared to neutral expressions; b) the implicit observation of pain expressions during time production modulated activity in the right MTG; c) functional connectivity between SMA and several cortical regions was correlated to the produced time interval during painful facial expression processing; d) neural activity of right MTG was positively related to the activity of regions belonging to the timing network, whereas it was negatively related to the activity of cortical regions involved in temporal decision making and in processing pain-related information.

*Mechanisms underlying time underestimation*. According to the internal clock theory, subjective experience of time can be differently modulated by attention and arousal. Increasing arousal is thought to speed up the pacemaker rate, leading to temporal overestimation [19–21]. Previous EEG and TMS studies suggest that negative emotions trigger action preparation [61,62], and several authors [8] hypothesize that temporal overestimation during perception of emotional faces (angry and fearful) may facilitate action in potentially threatening situations. Attention is thought to control the switch: when attentional resources are divided during timing, some pulses are lost because of the flickering of the switch or the prolonged latency of its closure, thus causing temporal underestimation [29,30]. Conflicting results on time estimates were reported by previous research using noxious stimulations [10–12,15]. However, actual noxious stimulation is conceivably more arousing than the observation of facial expressions of pain, especially when the latter are implicitly processed; therefore, the behavioral effect of observing pain expressions revealed in our experiment may not be necessarily similar to what happens during actual pain perception.

According to a previous study [3], unpleasant pictures are underestimated in low arousal conditions, whereas they are overestimated in high arousal conditions. There is the possibility that our stimuli were not very arousing and that their emotional valence diverted attention away from time, thus causing underestimation. It should be underlined that we neither collected subjective, behavioral or physiological measures of arousal, nor quantified the attention-catching effect of painful faces during our fMRI study. Therefore, we can not directly assess whether the observed temporal distortions were mainly related to attention or arousal effects. At this regard, it would be interesting to evaluate the effect of painful facial expression using other temporal tasks; indeed, previous studies showed different modulations on time judgments as a function of the task used [63]. Namely, the temporal bisection task, which has commonly been used to study time perception [64–67] and the effect of facial emotional expression on time [28,68,69], may be better suited for disambiguating mathematically between arousal-based and attentional effects, since arousal-related changes are multiplicative [9], whereas attentional effects are additive [70]. Finally, a limitation of the study was that only one emotional facial expression (i.e., painful) was used. Consequently, it is not possible to demonstrate whether the attention-catching effect of the emotional expression was specifically due to pain or to the greater intensity of the expression compared to neutral faces. In particular, the comparison between pain and disgust should be taken into account in future studies, because the experience of disgust increases pain sensitivity [71], and because pain and disgust share a common neural pattern of activity in various cortical regions [72].

*Modulation of emotion- and time-related neural circuits*. Our fMRI results show the functional modulation of right MTG when comparing implicit pain-vs.-neutral expression processing during temporal production. Previous studies showed that this region belongs to the cortical network subserving emotional face processing [35,50,51] and contributes to decoding the emotional meaning of painful faces [38]. Because the anterior insular cortex is activated during both temporal processing and emotional experience [73], Craig (2009) suggests that emotion-induced distortions of time may depend on insular activity. Indeed, the only two previous neuroimaging experiments [4,40] examining the neural mechanisms of emotional modulation of time revealed activity in the insular cortex, but also in other regions belonging to the network typically engaged during timing. The first study showed that the incorrect judgment of an aversive image as lasting longer than a non-aversive picture was associated with modulation of neural activity in the amygdala, putamen and insula [4]. The effect of facial emotion on neural activation within the timing network was explored in another study, further demonstrating the tendency to overestimate the duration of angry and happy faces when compared to neutral expressions; emotional expressions were shown to modulate the activity of SMA, IFG and AI [40]. In both studies, functional data resulted from analyses implemented within a-priori selected regions of interest belonging to the timing network, and did not take MTG into consideration.

We hypothesize that time processing alterations related to the perception of painful faces may rely on the interaction between the neural substrates of pain expression processing and the cerebral timing network. Our first PPI analysis suggests that MTG may be the key region mediating the interplay between these two neural systems.

Indeed, as expected, the activity of right MTG was positively related to neural activity within regions belonging to the timing network and mediating the clock stage [49,53,57,74,75], including: a) bilateral putamen, caudate nucleus and globus pallidus, sub-cortical regions engaged in formulating representations of time; b) right AI, IFG and cerebellum, areas involved in the accumulation of the pulses; c) primary sensorimotor cortex, commonly activated during temporal production tasks; d) bilateral SMA. A recent meta-analysis showed that SMA contains the largest number of significant voxels across all timing studies, suggesting that this region may be part of the putative clock mechanism as temporal accumulator [49]. Our results show that neural activity of this region is mainly related: i) to the activation of right IFG/right AI and cerebellum during implicit pain processing, and ii) when longer intervals are produced, to the activation of bilateral orbito-frontal cortex, medial superior frontal gyrus, left ventro-lateral prefrontal cortex, ACC and right angular gyrus.

Moreover, our results show a negative correlation between the neural activation of right MTG and of bilateral dlPFC. This prefrontal area is thought to be recruited during the decision-making stage: when time evaluation is required, the activation of this region mediates the comparison between the estimated interval and the abstract temporal references, thus allowing the response selection [75]. Our data suggest that this decisional process may be hindered by neural activity related to emotional processing.

Finally, our functional analyses showed that increasing activity of MTG was related to decreasing activity within several cortical regions involved in pain perception [59,76,77] as well as in the observation of painful stimulations and facial expressions of pain [36,38,39,60,78–80], such as posterior insula, ACC, primary and secondary somatosensory cortex.

In summary, our findings demonstrate that the activation of a region decoding emotional meaning (posterior MTG) is positively related to cortical areas mediating the clock stage, whereas it is negatively related to key regions involved in the temporal decision-making stage and in processing pain-related information. We can speculate that, at the beginning of the interval to be produced, attention was automatically captured by the expression of pain, thus reducing attentional resources available for the temporal task, interfering with the closure of the switch and causing the loss of some pulses. Therefore, the resulting underestimation of the interval may depend on the concurrent engagement of regions subserving implicit processing of painful expressions and of cerebral areas that mediate emission and accumulation of pulses, but also temporal decision-making.

## Conclusions

The present study provides the first evidence that observation of facial expressions of pain interact with timing at the behavioral and neural level. The right posterior MTG appears to be the key region mediating the interaction between the brain network analyzing painful expressions and the neural network involved in timing.
